# Supplementary figures and images for: The Landscape of Presence/Absence Variations during the Improvement of Rice
Source: Genes (Basel). 2024 May 19;15(5):645. doi: 10.3390/genes15050645 (PMC11120952; doi:10.3390/genes15050645)

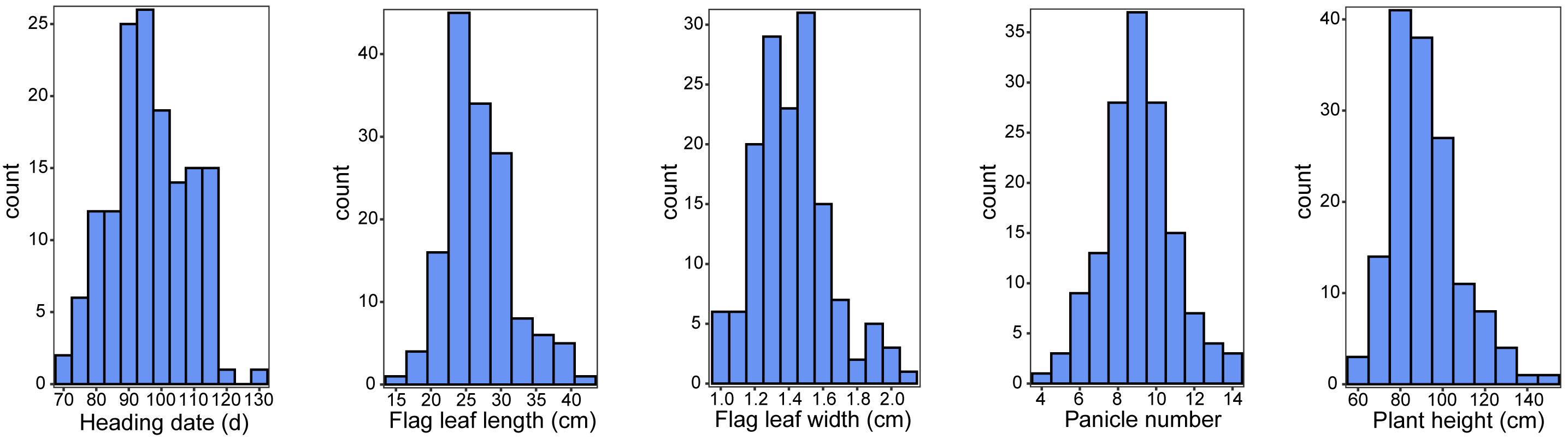

Supplement: Supplementary file 1 [file genes-15-00645-s001.zip › Figure S1.tif]

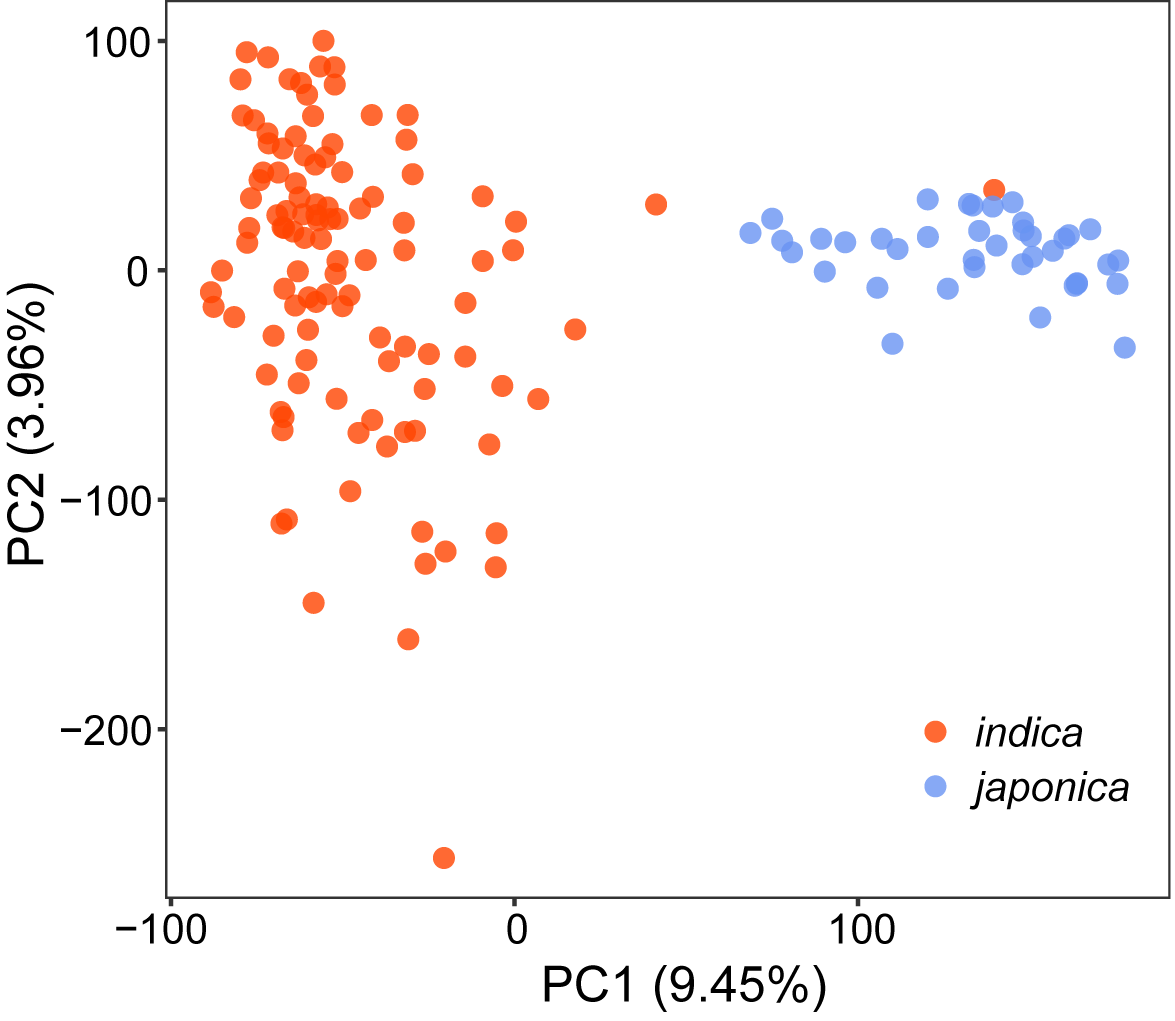

Supplement: Supplementary file 1 [file genes-15-00645-s001.zip › Figure S2.tif]

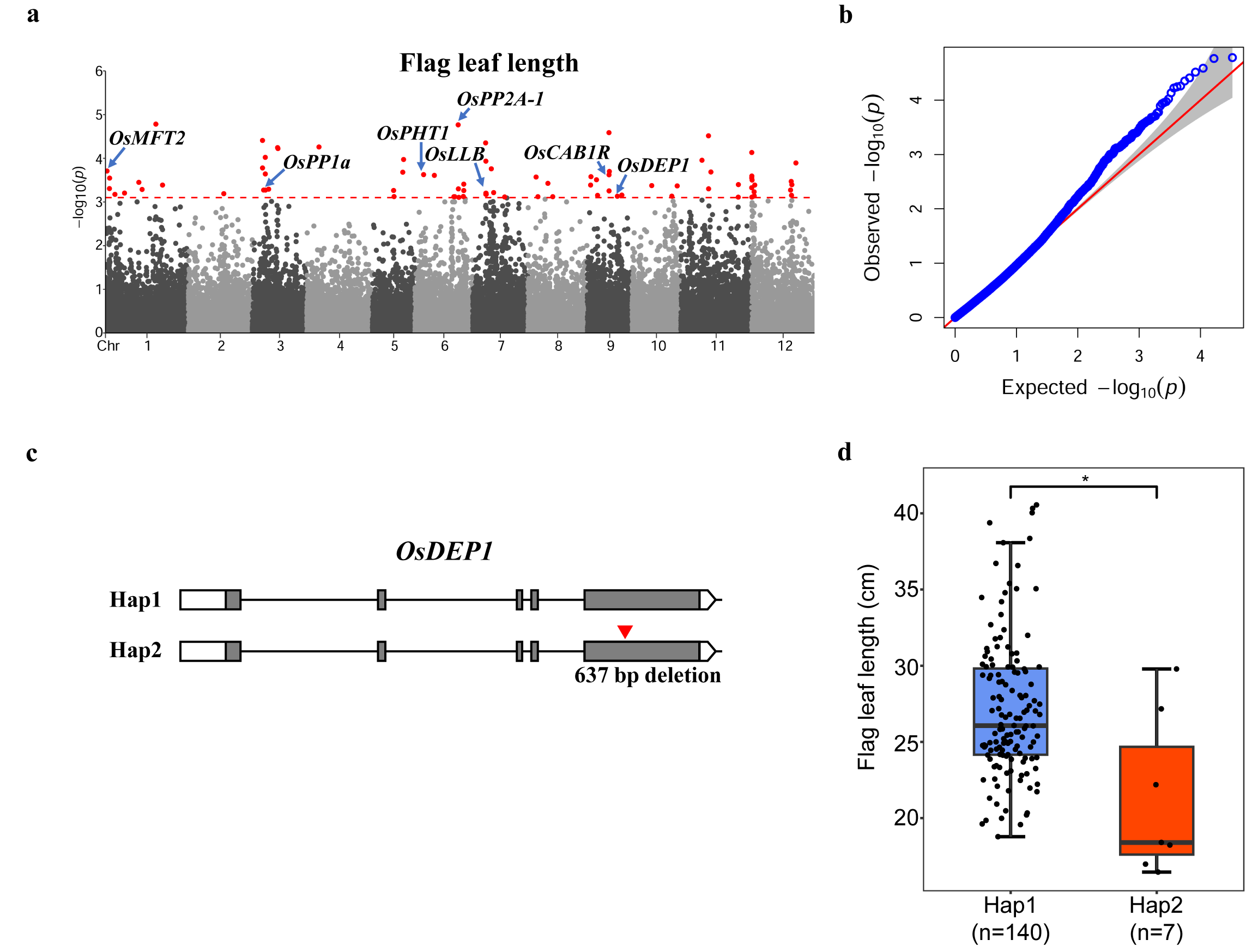

Supplement: Supplementary file 1 [file genes-15-00645-s001.zip › Figure S3.tif]

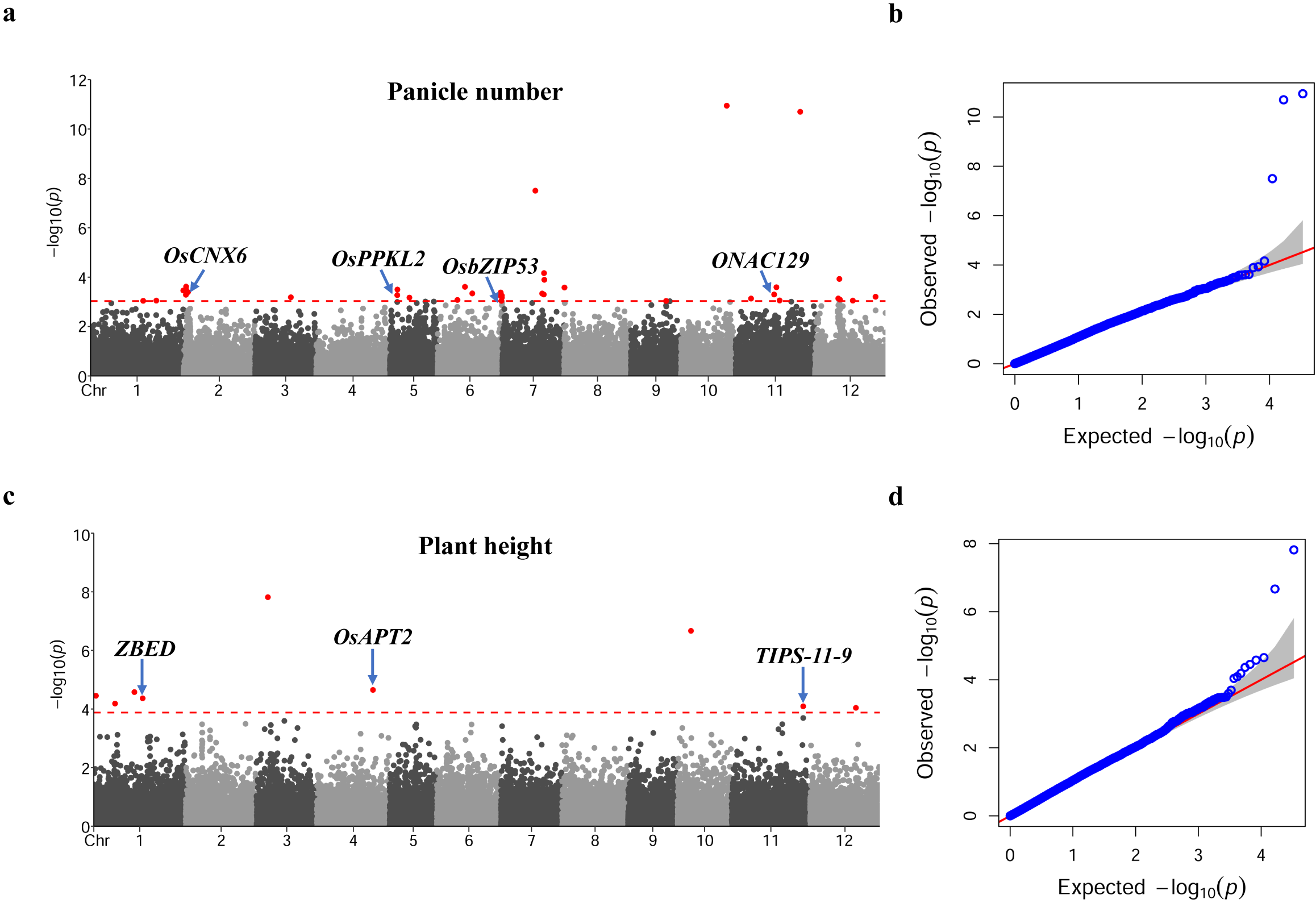

Supplement: Supplementary file 1 [file genes-15-00645-s001.zip › Figure S4.tif]

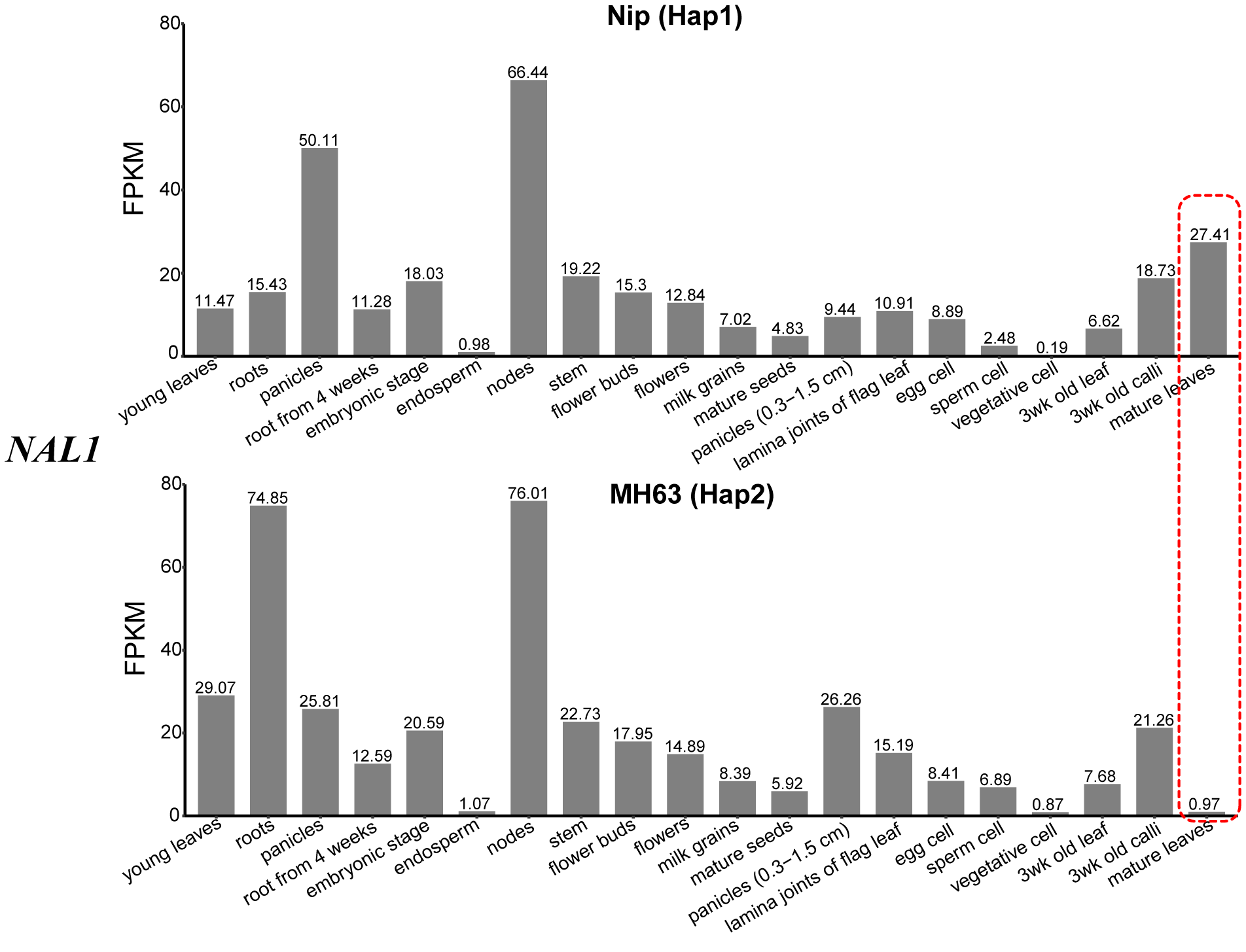

Supplement: Supplementary file 1 [file genes-15-00645-s001.zip › Figure S5.tif]
